# Failure of Dental Operations

**Published:** 1903-05-15

**Authors:** F. L. Platt


### FAILURE OF DENTAL OPERATIONS.

BY F. L. PLATT, D.D.S.

Throughout the range of recent dental literature, as it is represented by contributions to our dental journals, it is pleasing to note the presence of an optimistic spirit forever calling attention to the wonderful "progress of dentistry." Occasionally some intrepid writer with the courage of his convictions apparent in every paragraph of his production calls attention, not necessarily in a spirit of pessimism, to the frailties of the dental profession and the failures of our operative procedures.

Both classes of writers to whom reference is made are to be commended for the attitude they take, for the optimistic school of contributors to our journals, those who describe in glowing terms the invariable success of certain operations, are the ones who stimulate the spirit of emulation and lead others out of the well-beaten paths they might otherwise forever tread, while the more cautious, thoughtful, observing class of writers, who see and are not afraid to mention defects in their own and others' work, encourage research, promote intelligent thought and foster a spirit of caution in many who otherwise might be led into serious error by their over-enthusiastic friends.

To the latter class of writers belong those who see in dentistry a brilliant present and a more glorious future; those who would set every step of dental progress on a sound foundation and "make haste slowly" as the old adage goes, by determining the cause of failure, before trying to remedy it by new and unproven methods and untried devices.

In the minds of these members of our profession there seems to be ever present the thought that, no matter who performs the operation or what method may be employed, there is always to be taken into consideration the fact that in the great majority of cases it will sooner or later fail. This is a sad but true statement of facts as we know them to exist. We glibly style our operations temporary or permanent, as the case may be, yet on sober second thought we must reluctantly admit that most of them should be in the former rather than in the latter class.

Some studious gatherer of statistics has said that the average life of a gold filling is three years. Should such work be called permanent?

Amalgam, that much abused and rarely well used material, is said by some to be but a sorry excuse for a filling-material, while others give amalgam fillings a period of longevity not easily determined in a single human lifetime. But amalgam fillings fail, and so do all sorts of materials and all classes of operations. Is it not pertinent then to ask, "Why?" Does it not seem a melancholy comment on dental skill that failure is to be forever in sight? Are we as a profession doing all we may to secure better results for our daily toil and teaching those who are just entering professional life all they should be taught? Let us review the situation as we know it to exist and briefly consider the failures of dental operations.

No sounder rule for the guidance of medical and dental practitioners was ever laid down than that given by the late Professor Garretson to his students when he said: "Would you cure disease? Remove the cause."

It is not the purpose of this paper to review the causes of dental decay or of its recurrence, but they may be briefly stated to be a "non-cognizable systemic influence," possibly another name for a hereditary tendency, by virtue of which some teeth are prone to decay and others are practically immune, and the effect of environment, both of the individual and his teeth. A clean, healthy and remunerative occupation, pleasant surroundings and agreeable social conditions for the individual, and a clean, healthy, well-cared-for mouth for his teeth, are conditions of environment which will exert an opposite influence, so far as dental caries is concerned, to conditions representing the natural result of ignorance and idleness.

With the secondary causes of decay, bacterial and chemical action, we have nothing to do within the limits of this essay; they are included in the generalization of environment, but is it not apparent that to reach the fundamental cause of the failure of dental operations we must first consider the original causes which necessitated their performance? Then unless these are removed we need not expect to see an operation, purely mechanical in nature, correcting evil conditions which are inherent in the individual or his surroundings.

Primarily, then, to prevent the failure of dental operations, the conditions enumerated must be improved or totally changed, for the recurrence of decay is but to be expected when its original cause is continually active.

Many reasons may be given for the failure of dental operations which may be said to lie either in the quality of the material employed to replace lost tooth structure, in the operation itself or in the manner of its performance.

There seems to be an unfortunate tendency on the part of the public and many members of our profession to demand cheap operations and cheap materials. We seem to be living in an age of cheap things; in an age when millions of people value a thing simply by its cost in dollars and cents and not by its actual worth in services rendered and objects obtained. To meet this demand dealers vie with one another to produce the cheapest alloy, and dentists struggle for supremacy in inserting fifty cent fillings. Is it a wonder such operations fail?

Many thousands of dental operations fail because they are not indicated in the places where they are employed. There seems to be a lack of discrimination on the part of many operators and an inability to select for each individual case the methods and the materials best suited to its conditions. Operators are either ignorant of the individual qualities of their materials and the conditions indicating their use, or else are guided as they may also be in the purchase of their materials by the financial consideration involved in each transaction. So, many gold fillings are placed in cavities where amalgam would give better results, and bridges without number are placed on piers unable to bear them.

Leaving all other considerations aside, it is highly probable that the most frequent cause of the failure of dental operations lies not so much in the conditions already mentioned as in the manner in which the operations are performed. Some one has said that "Time is the stuff of which worlds are made," and no one knows its value better than the average dentist.

Early in his career he has found out just how much he must make every day to meet his expenses; he has learned that people expect cheap work, and rather than disappoint them he meets their demands and gives them cheap work—cheap in every sense of the word. In doing this he has increased the number of operations he must do every day to live; inevitably he must slight his work. Cavities must be poorly prepared and fillings left unfinished; plates and bridges are poorly fitted and diseased conditions must be inadequately treated; all because the fee he expects to receive is not sufficiently large to allow him to take time to do his work well—not even as well as he may know how to to do it. Is it a wonder that such work fails? Is it strange that working under such self-imposed conditions the average operator has no time to study the causes of decay and the best means to combat it, and still has time to put his theories, if he has any, into practice?

It is not the purpose of this paper to draw a gloomy picture of modern dentistry, but only to call attention to what seems to be the chief cause of the failure of dental operations. Do you ask a remedy? Teach your patients that you do not sell materials, but skill; that time is the essence of every contract you undertake, and that time is the most valuable thing in the world, the only thing except life, which once lost cannot be recovered.

Teach those just entering our profession that competition with cheap men must not be in prices, but in the quality of the services rendered, and that no single operation must be slighted, even in the smallest degree, and no patient left uninstructed in the duty he owes himself and his dentist in the daily care and personal examination of his teeth.—*Pacific Gazette.*

